# An omnigenic interactome model to chart the genetic architecture of individual plants

**DOI:** 10.1093/hr/uhaf345

**Published:** 2025-12-16

**Authors:** Changjian Fa, Guijia Wang, Wenqi Pan, Yu Wang, Jincan Che, Ang Dong, Dengcheng Yang, Rongling Wu, Shing-Tung Yau, Lidan Sun

**Affiliations:** Center for Computational Biology, School of Grassland Science, Beijing Forestry University, Beijing 100083, China; Beijing Key Laboratory of Topological Statistics and Applications for Complex Systems, Beijing Institute of Mathematical Sciences and Applications, Beijing 101408, China; State Key Laboratory of Efficient Production of Forest Resources, Beijing Key Laboratory of Ornamental Plants Germplasm Innovation and Molecular Breeding, National Engineering Research Center for Floriculture, School of Landscape Architecture, Beijing Forestry University, Beijing 100083, China; Beijing Key Laboratory of Topological Statistics and Applications for Complex Systems, Beijing Institute of Mathematical Sciences and Applications, Beijing 101408, China; Beijing Key Laboratory of Topological Statistics and Applications for Complex Systems, Beijing Institute of Mathematical Sciences and Applications, Beijing 101408, China; Center for Computational Biology, School of Grassland Science, Beijing Forestry University, Beijing 100083, China; Beijing Key Laboratory of Topological Statistics and Applications for Complex Systems, Beijing Institute of Mathematical Sciences and Applications, Beijing 101408, China; Beijing Key Laboratory of Topological Statistics and Applications for Complex Systems, Beijing Institute of Mathematical Sciences and Applications, Beijing 101408, China; Beijing Key Laboratory of Topological Statistics and Applications for Complex Systems, Beijing Institute of Mathematical Sciences and Applications, Beijing 101408, China; Program in Applied Statistics, Shanghai Institute for Mathematics and Interdisciplinary Sciences, Shanghai 200433, China; Beijing Key Laboratory of Topological Statistics and Applications for Complex Systems, Beijing Institute of Mathematical Sciences and Applications, Beijing 101408, China; Program in Applied Statistics, Shanghai Institute for Mathematics and Interdisciplinary Sciences, Shanghai 200433, China; Yau Mathematical Sciences Center, Tsinghua University, Beijing 100084, China; State Key Laboratory of Efficient Production of Forest Resources, Beijing Key Laboratory of Ornamental Plants Germplasm Innovation and Molecular Breeding, National Engineering Research Center for Floriculture, School of Landscape Architecture, Beijing Forestry University, Beijing 100083, China

## Abstract

Complex traits are controlled by many unknown genes, making it difficult to elucidate a global picture of the genotype–phenotype map. Here, we develop a statistical mechanics model to contextualize all possible genes into informative, dynamic, omnidirectional, and personalized idopNetworks. This model, derived from the combination of functional mapping and evolutionary game theory, can visualize and trace how genes act and interact with each other to shape the genetic architecture of complex traits. The model can estimate changes in the genotypic value of one gene due to the influence of other genes, specifically on individual subjects, surpassing traditional quantitative genetic studies that can only capture the marginal effect of a gene at the population level. We reconstruct growth idopNetworks from a genome-wide mapping data in a woody plant, mei, identifying unique genetic interaction architecture that distinguishes between fast-growing trees and slow-growing trees. We perform computer simulation to validate the statistical power of the model. IdopNetworks can disentangle the genetic control mechanisms of complex traits and provide guidance on how to alter phenotypic values of specific individuals by promoting or inhibiting the expression of interactive genes.

## Introduction

Given their paramount importance to agriculture, biomedicine, and evolutionary biology, the genetic architecture underlying quantitatively inherited traits has been an important focus and interest of research in life science [1–4]. Despite worldwide tremendous effort to resequence the genomes of a wide variety of species, we still know little about how many loci contribute to trait variation, how they function, and where they reside in the chromosomes [5]. This inadequacy of knowledge may be due to the limitation of result explanations by current quantitative genetic analysis approaches. Most commonly used genome-wide mapping or association studies on the basis of a reductionist thinking attempt to detect significant associations between the phenotype and the genotype at single Single Nucleotide Polymorphisms (SNPs) [6, 7]. This analytical strategy can only estimate marginal (i.e. overall) effects of a gene without taking into account how this gene interacts with other genes to determine the phenotype. For example, some genes are detected to be significant, but their significance may not be attributed to their intrinsic function, rather arise from the extrinsic promotion of other genes for them. On the other hand, if genes are tested to be insignificant, this may not necessarily mean that their intrinsic effects are not important, but they are canceled out by negative regulators. In this sense, it is crucial for revealing the interactive relationships among different genes to gain a mechanistical understanding of genetic control [8, 9].

Another significant drawback of current genetic analysis is that it estimates genetic effects at the population level, but ignores interindividual variation, which is particularly useful for plant and animal breeding aimed to select superior genotypes of high productivity based on their average performance. However, it is largely infeasible for many other applications, such as human genetics, in which case each and every individual should be considered and treated differently. In practice, among three possible genotypes at a significant locus, plant or animal breeders may only choose one that reaches the highest mean productivity, no matter how individuals within this genotype vary. For plant breeders or human geneticists, interindividual variation within the same genotype at a locus of interest due to the epistatic effects of other loci can guide them to design precise breeding programs or individualized intervention strategies. For example, two individuals have the same genotype *AA* at locus 1, which carry genotypes *BB* and *Bb* at locus 2, respectively. If the influence of *BB* on *AA* for an individual differs from that of *Bb* on *AA* in the other individual, then different strategies for genetic manipulation should be used to maximize the genotypic values of *AA* for these two individuals. Such information cannot be obtained from traditional population genetic analysis.

The above two limitations of current quantitative genetic analysis can be circumvented by combining functional mapping and evolutionary game theory, two previously disjointed concepts from different disciplines. Functional mapping is a statistical approach for mapping dynamic genes that govern developmental trajectories of complex traits [10–13]. The central tenet of this approach is to contextualize mathematical equations of organismic growth and development into a statistical mapping setting, which allows the time-varying genetic effect of a gene to be characterized. Functional mapping has been instrumental for estimating and understanding the genetic control of complex traits in plants and other organisms [14–20]. Evolutionary game theory [21] is the combination between game theory, originated from economic studies [22, 23], and evolutionary biology. This theory can explain how a player maximizes its payoff by adopting an optimal strategy derived from the strategies of its counterparts without need of rationality assumption. Sun *et al*. [24] derived a system of mixed ordinary differential equations (mODEs) to decompose the overall payoff of a player into the independent component resulting from its own strategy and dependent component due to the strategy of its counterparts. We encode estimates of independent components as nodes and those of dependent components as edges into mathematical graphs, giving birth to the reconstruction of informative, dynamic, omnidirectional, and personalized networks (idopNetworks) [25–30].

Here, we leverage Sun *et al*.’s [24] mODE model into an SNP effect-based generalized statistical mechanics framework. Under this framework, marginal effects of each SNP estimated by functional mapping are decomposed into independent genotypic values and dependent genotypic values through the lens of evolutionary game theory. By coalescing these independent and dependent components into idopNetworks, this model framework provides a unique avenue to chart the complete genetic architecture of complex traits by illustrating the roadmap of how each SNP interacts with every other SNP. This model can capitalize on a complete set of SNPs from the whole genome to form multilayer interactome idopNetworks. More importantly, different from previous models that infer an overall network from a population, the new model can reconstruct idopNetworks for individual samples based on genotypic values of each SNP. This information is crucial for precision medicine and precision breeding. We implement this model to analyze a genome-wide mapping dataset from a full-sib family of an ornamental woody plant—mei (*Prunus mume*) [31, 32], gaining different insight into the genetics of growth traits.

## Results

### Bivariate functional mapping

Two different mei varieties, Liu Bandan (as the female parent) and Sanlun Yudie (as the male parent), were crossed to generate an F_1_ progeny population F-2015 [33]. The genomes of 184 F_1_ individuals from this progeny population were resequenced throughout the genome, producing 5393 segregating SNPs, including 3986 testcross markers and 1407 intercross markers [34, 35], distributed over eight chromosomes. Scions from the seedlings of this population were grafted on 5-year rootstocks of healthy mei trees in wintertime at the Experimental Station of Beijing Forestry University Center for Computational Biology, located in Nantong, Jiangsu Province, southeast China. In the coming spring, scions sprout into shoots. Ten randomly chosen shoots from each progeny were measured for their heights and diameters at base once every 2 weeks, starting at 1 week since sprouting and ending when trees stop their growth in the fall.

**Figure 1:**
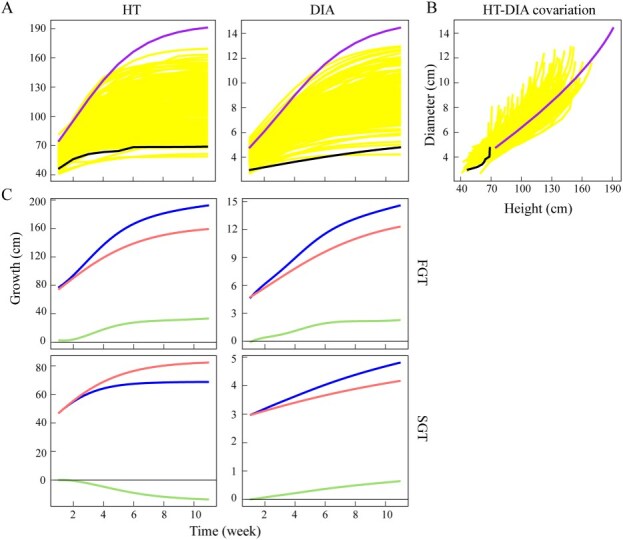
Growth analysis. (A) Shoot height (HT) and shoot diameter (DIA) growth trajectories of individuals (yellow line) from an F_1_ mei mapping population. An FGT (purple line) and SGT (black line) are chosen for subsequent analysis. (B) Covariation of shoot height growth against shoot diameter growth over ontogeny. (C) Systems mapping of shoot height growth and diameter growth for the FGT and SGT. The overall growth curve of a trait (blue line) is decomposed into independent component curve (red line) and dependent component curve due to the influence of the other trait (green line).

Growth trajectories of shoots, which can be fitted by growth equation (2), display tremendous variation among individuals, from which a large and small F_1_ progeny are identified as representatives of a fast-growing tree (FGT) and a slow-growing tree (SGT), respectively (Fig. 1A). By plotting shoot heights over stem diameters, we find that these two traits covary in a nonlinear manner, with the pattern of covariation displaying substantial interindividual variation (Fig. 1B). We use systems mapping [12, 13] to characterize how shoot height and diameter growth are mutually influenced by each other. As illustrated by Fig. 1C, these two traits are mutually promoted for the FGT, but for the SGT, shoot height growth is repressed by shoot diameter growth whereas the latter is promoted by the former.

**Figure 3:**
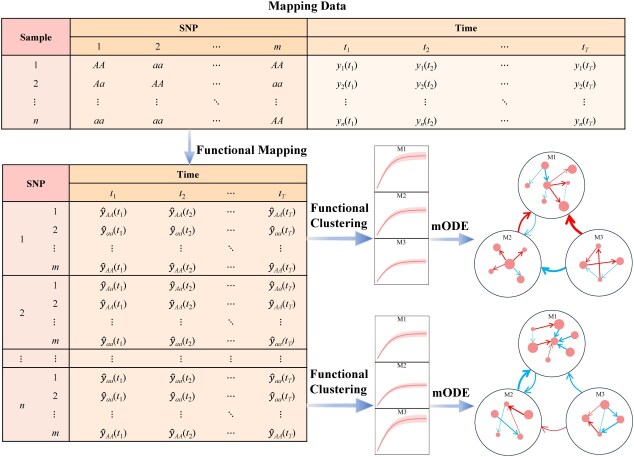
Data format for functional mapping (FunMap) with *m*-SNP genotypes and growth traits measured at *T* time points for *n* samples. FunMap estimates growth parameters for each genotype and uses them to predict its time-varying genotypic values for each SNP. Functional clustering (FunClu) is implemented to classify all SNPs into multiple modules based on their similarity of genotypic curves. The mODE is formulated to reconstruct multilayer idopNetworks for each individual sample, whose upper layer is expressed as module–module interaction coarse-grained networks and whose lower layer is expressed as SNP–SNP interaction networks.

**Figure 4:**
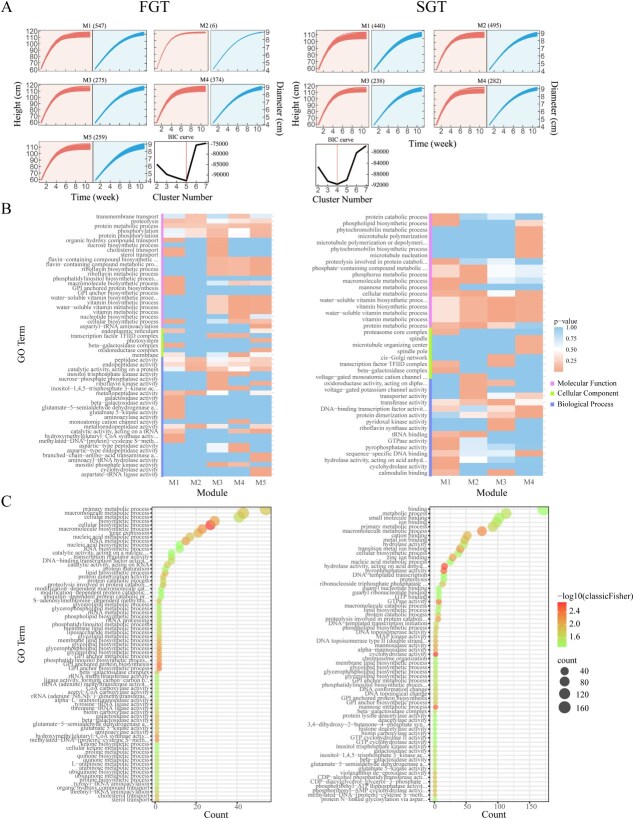
BiFunClu of all SNPs based on their genotypic values in shoot height and diameter cogrowth trajectories for the FGT and SGT. (A) Five modules (M1–M5) detected according to BIC values. (B) GO analysis of genes from each module. (C) GO analysis of gene distribution from M1.

**Figure 2:**
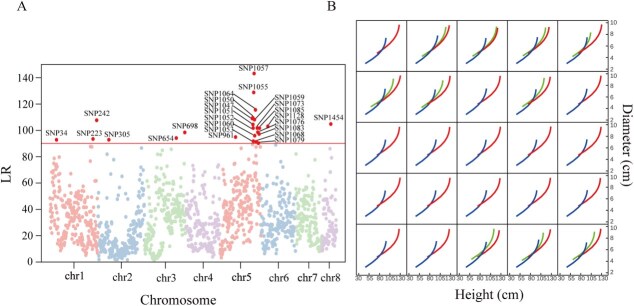
Genome-wide functional mapping. (A) Manhattan plot of log-likelihood ratios (LR) as a test statistic for all SNPs throughout the eight mei chromosomes. Those marked SNPs, whose LR are beyond the critical threshold determined from permutation tests, are considered to be significant (coined QTLs) for shoot height–diameter cogrowth. (B) Shoot height–diameter cogrowth ontogenic curves for three genotypes at intercross QTLs and two genotypes at testcross QTLs.

We implement bivariate functional mapping (biFunMap) [36, 37] to estimate the growth parameters of shoot heights and diameters for different genotypes at each SNP. By testing how genotypes at a locus differ in height–diameter cogrowth trajectories from each other, we identify 25 significant SNPs distributed on various chromosomal positions associated with both height and diameter growth trajectories (Fig. 2A). We draw height–diameter relationship curves for each genotype at these significant SNPs (Fig. 2B). Growth pattern in terms of height–diameter covariation varies among different genotypes at the same SNP, and these genotypic differences vary among SNPs. Gene annotations show that 21 of these SNPs reside in the adjacent regions of candidate genes. For example, SNP1052 is located in the adjacent region of the CESA3 gene, which encodes cellulose synthase A catalytic subunit 3, a protein considered essential for cellulose deposition in the primary cell wall [38]. SNP1057 resides within the NAXT1 gene encoding Nitrate Excretion Transporter 1, a protein involved in passive nitrate efflux [39, 40]. These genes are not only associated with processes directly linked to nitrate, such as transport, reduction, and assimilation, but also are involved in the metabolism of amino acids, nucleotides, and other nutrients. Additionally, these genes are associated with hormone biosynthesis and signaling, as well as regulatory genes, including kinases, phosphatases, and transcription factors (TFs) that play a role in growth and development [41–44].

### Bivariate functional clustering

To test how different SNPs interact with each other to mediate the pattern of height–diameter covariation, we first calculate genotypic values for each individual carrying a specific genotype at each SNP using growth parameters estimated by biFunMap (Fig. 3). In principle, using these estimated growth data, we can reconstruct idopNetworks for every individual. Without loss of generality, we choose a big tree (FGT) and a small tree (SGT) from the mapping population to compare growth-related differences in idopNetwork architecture. We first implement bivariate functional clustering (biFunClu) [45] by integrating shoot height and diameter growth to classify all SNPs, based on their similarity of time-varying genotypic values, into five modules for the FGT and four modules for the SGT (Fig. 4). The majority of significant SNPs are attributed to module M1, 72% for the FGT and 84% for the SGT. The nonrandom distribution of significant SNPs across modules suggests that they may function in a similar manner. We perform Gene Ontology (GO) analysis of SNPs within the module and find that modules vary dramatically in terms of molecular functions, cellular components, and biological processes. Module M1 of both FGT and SGT is enriched in the beta-galactosidase complex, which plays a role in plant growth by regulating cell wall metabolism [46] and protein and RNA metabolism. Module M1 of the FGT is additionally enriched with genes encoding S-adenosylmethionine-dependent methyltransferase, N-methyltransferase, and hydroxymethylglutaryl-CoA synthase, whereas M1 of the SGT contains genes associated with mannose metabolism, cyclohydrolase activity, pyrophosphatase activity, and hydrolase activity, particularly those targeting phosphorus-containing anhydrides. In general, M1 of both FGT and SGT contains genes important for plant growth, but with significant differences between the two trees.

**Figure 5:**
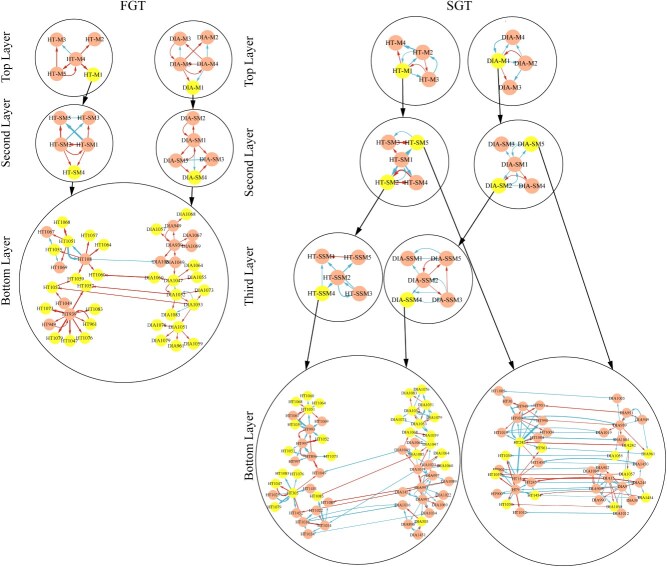
4MidopNetworks mediating shoot height and diameter growth for the FGT and SGT. At the top layer are the module–module interaction networks and at the bottom layer are the SNP–SNP interaction networks. Red and blue arrowed lines represent positive and negative regulation, respectively, with the thickness of lines proportional to the strength of regulation.

**Figure 6:**
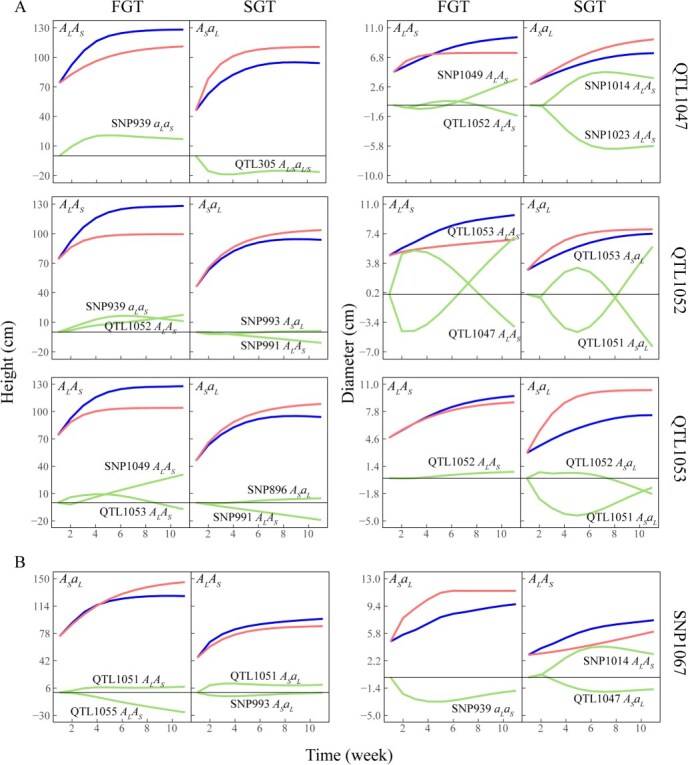
The decomposition of overall genotypic curves (blue line) for a genotype at each of three QTLs (A) and an insignificant SNP (B), carried by the FGT and SGT, into independent genotypic curves (red line) and dependent genotypic curves due to the influence of loci (green line). Shoot height and shoot diameter growth display different patterns of decomposition. SNP alleles are denoted by A and a, subscribed by the initials of female parent Liuban Dan (L) and male parent SanlunYudie (S).

**Figure 7:**
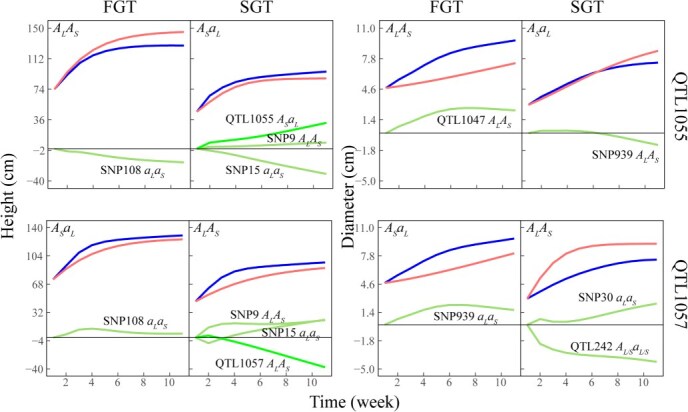
The decomposition of overall genotypic curves (blued curves) into independent genotypic curves (red lines), epistatic dependent genotypic curves (light green lines), and pleiotropic dependent genotypic curves (dark green lines). Allelic notation is given in Fig. 7.

**Figure 8:**
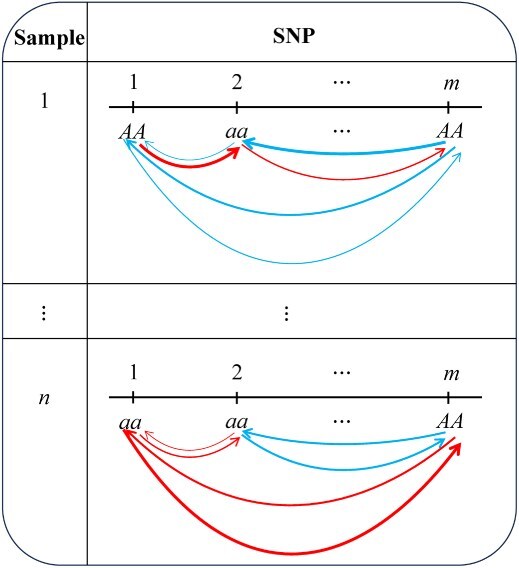
Diagram of bidirectional, signed, and weighted epistasis between genotypes at different SNPs, expressed in individual samples. Red and blue arrowed lines represent positive and negative regulation of one genotype by the other genotypes, respectively, with the strength of regulation proportional to the thickness of lines.

### Multilayer interaction networks

We classify all SNPs as a whole into distinct modules and further classify each module into distinct submodule and each submodule from module into sub-submodules. This process repeats until the number of SNPs within a unit reduces to a level at which all SNPs can maintain stable mutual relationships. We reconstruct interaction networks at different levels from modules all the way down to SNPs, forming a multilayer, multiplex, multispace, and multifunctional idopNetworks (4MidopNetwork) mediating both shoot height and diameter growth for the FGT and SGT, respectively. We find that directional regulation serves as the primary internal working of SNP–SNP networks, with 85% vs 15% of regulations being positive and negative for the FGT but 38% vs 62% being positive and negative for the SGT (Fig. 5). This probably implies that the high frequency of gene promotion is beneficial for better shoot growth. The names of modules, submodules, and SNPs are prefixed by HT and DIA to distinguish between the height network and diameter network. SNPs HT108 and HT939 in the height network of the FGT play a leading role, regulating six and seven distinct SNPs, respectively. HT108 is located in the gene of the subtilase (SBT) family controlling plant growth and development by cleaving the phytosulfokines PSK3, PSK2, and PSK5 *in vitro*, plant growth factors or peptide hormones that promotes plant cell differentiation, organogenesis, somatic embryogenesis, and cell proliferation [47]. HT939 resides in the gene-encoding ubiquitin-specific protease UBP12, a positive regulator of root meristem development [48]. Interestingly, for the height network of SGT, hubs have shifted to different SNPs.

Unlike significant SNPs, referred to as Quantitative Trait Loci (QTLs), in height networks that tend to be regulated, most QTLs in diameter networks tend to regulate other loci, with four of them (DIA1047, DIA1051, DIA1052, and DIA1053) acting as hubs for the FGT. DIA1047 is in the proximity of genes encoding basic helix–loop–helix proteins, which are TFs that regulate root development. DIA1051 is located near genes encoding Delta-1-pyrroline-5-carboxylate synthase, which plays a key role in proline biosynthesis and contributes to osmoregulation in plants. DIA1052 is positioned adjacent to genes encoding the probable catalytic subunit of cellulose synthase terminal complexes, which is required for beta-1,4-glucan microfibril crystallization, a major mechanism of cell wall formation. DIA1047 regulates four QTLs for the FGT, whereas it regulates two QTLs for the SGT. In summary, alterations in interactions between hub SNPs/QTLs and other QTLs are instrumental for shaping shoot growth.

While FunMap can identify QTLs for stem growth, idopNetworks can mechanistically explain how these QTLs function by decomposing the genotypic growth curve of a QTL into its independent genotypic growth curve and dependent genotypic growth curve due to the influence of other SNPs on it (Fig. 6A). SNP1047 is a QTL detected to be associated with height–diameter covariation (Fig. 1A), which inherits maternal alleles from parent Liu Bandan (with *A_L_A_L_*) and paternal alleles from parent Sanlun Yudie (with *A_S_a_S_*) to generate two genotypes *A_L_A_S_* and *A_S_a_L_* corresponding to the FGT and SGT, respectively. We find that these two genotypes display a similar amount of independent genotypic growth in shoot height, but genotype *A_L_A_S_* is promoted by genotype *a_L_a_S_* at SNP939, leading to the former’s high genotypic value in the height growth of the FGT, whereas genotype *A_S_a_L_* is repressed by genotype *A_L/S_a_L/S_* at SNP305, making the former’s observed height growth below the independent genotypic value for the SGT (Fig. 6A). Note that *A_L/S_a_L/S_* means that this heterozygous genotype has alleles with an unknown parental origin. In this sense, by knocking off negative regulate, genotype *A_L/S_a_L/S_* of SNP305, genotype *A_S_a_L_* for the SGT can be independently expressed, thus its height growth can increase. The influence of this SNP on diameter growth can also be explained in a meaningful way, although the diameter growth of both FGT and SGT is regulated by two SNPs (Fig. 6). We find that, if there was no interaction, genotypes *A_S_a_L_* at SNP1047 would display a greater rate of diameter growth than genotype *A_L_A_S_*, but the former is promoted by the net influence of SNP1049 and SNP1052 in the FGT, whereas the latter is repressed by the net influence of SNP1014 and SNP1023 in the SGT. It is interesting to find that genotype *A_S_a_L_* at SNP1047 is negatively regulated by genotype *A_L_A_S_* at SNP1023, to a considerable extent, for the smaller tree, which suggests that, by knocking off this negative regulator, the diameter growth of the SGT can significantly increase, even surpassing that of the FGT. A similar phenomenon is observed for many other QTLs (Fig. 6A).

SNP1067 is one of many insignificant SNPs through genome-wide testing using FunMap. However, we find that this SNP is intrinsically not insignificant for shoot diameter growth because its independent genotypic value is considerably higher for one genotype *A_L_a_S_* (carried by the FGT) than for the other genotype *A_L_A_S_* (carried by the SGT) (Fig. 6B). Because the genotype *A_L_a_S_* is inhibited by genotype *a_L_a_S_* at SNP939 whereas genotype *A_L_A_S_* is inhibited on the whole, the difference between these two genotypes is observed to be much shrunk. A similar trend, despite to a lesser extent, can be detected for the effect of SNP1067 on stem height growth.

### Wang’s entanglement networks

At the bottom of 4MidopNetworks, we link height epistatic networks and diameter epistatic networks through pleiotropic SNPs, forming expanded pleiotropic–epistatic webs that can characterize how different traits are governed not only by the same genes (pleiotropic genes), but also by the same SNP–SNP interactions (pleiotropic epistasis) [49]. These webs are called Wang’s entanglement networks because they are first reconstructed by Yu Wang and her team [50]. In Wang’s entanglement networks, pleiotropic regulations account for 7% of interactions for the FGT, whereas this number is as high as 15% for the SGT (Fig. 5). Different from its traditional definition, pleiotropy detected by our model is directional, signed, and weighted; i.e. the influence of shoot height on shoot diameter may be different from the influence of shoot diameter on shoot height in the extent and sign, which may be regulated by different genes. We find that among all pleiotropic effects, 25% is attributed to the influence of shoot height on shoot diameter, whereas 75% attributed to the influence of shoot diameter on shoot height. In addition, positive and negative regulations account for 75% and 25% for the FGT, but these numbers are 65% vs 35% for the SGT, suggesting that FGTs more frequently have positive height–diameter interactions than SGTs.

We decompose the overall genotypic values into independent genotypic values and dependent genotypic values including pleiotropic and epistatic effects (Fig. 7). We find that epistasis and pleiotropy play important roles in mediating the architecture of Wang’s entanglement networks. The shoot height growth of genotype *A_S_a_L_* at QTL1055 in the SGT contains negative epistatic regulation by SNP15’s *a_L_a_S_*, positive epistatic regulation by SNP9 *A_L_A_S_*, and positive pleiotropic regulation from shoot diameter growth. Pleiotropic regulation means that the genotypic value of one trait at a locus promotes or represses its genotypic expression in the other trait. The shoot height growth of genotype *A_L_A_S_* at QTL1057 in the SGT is negatively regulated by its shoot diameter growth. It should be pointed out that the shoot growth of the SGT is controlled not only by pleiotropy but also by epistasis. Taken together, individualized Wang’s entanglement networks and their dissection (Fig. 7) can systematically reveal tree-to-tree genetic differences in growth traits and their underlying allometric relationships.

## Computational simulation

Wang’s entanglement networks represent a mechanistic framework for understanding the genetic architecture of complex traits. To validate their statistical properties, we perform simulation studies under different scenarios. We assume that the genotypic value of an SNP is the sum of its independent genotypic value and dependent genotypic value, plus a normally distributed error. Consider a set of five interactive SNPs that affect a growth trait in Wang’s entanglement network, described by a system of mixed ordinary differential equations, expressed as


(1) \begin{equation*} \left\{\!\!\!\!\! \begin{array}{l}\frac{d{y}_1(t)}{dt}={Q}_1\left({y}_1(t):{\Phi}_1\right)+{Q}_{1\leftarrow 3}\left(y(t):{\Phi}_{1\leftarrow 3}\right)\kern11em \\ \, {}\frac{d{y}_2(t)}{dt}\!=\!{Q}_2\left({y}_2(t)\!:\!{\Phi}_2\right)\!+\!{Q}_{2\leftarrow 3}\left({y}_3(t):{\Phi}_{2\leftarrow 3}\right)\\ \qquad \quad \ \ +\ {Q}_{2\leftarrow{2}^{\ast }}\left({y}_{2^{\ast }}(t)\!:\!{\Phi}_{2\leftarrow{2}^{\ast }}\right)\\{}\frac{d{y}_3(t)}{dt}={Q}_3\left({y}_3(t):{\Phi}_3\right)+{Q}_{3\leftarrow 1}\left({y}_1(t):{\Phi}_{3\leftarrow 1}\right)\kern11.5em \\{}\frac{d{y}_4(t)}{dt}={Q}_4\left(\mathrm{y}(t):{\Phi}_4\right)+{Q}_{4\leftarrow 1}\left(\mathrm{y}(t):{\Phi}_{4\leftarrow 1}\right) \\ \qquad \quad \ \ +{Q}_{4\leftarrow{4}^{\ast }}\left({y}_{4^{\ast }}(t)\!:\!{\Phi}_{4\leftarrow{4}^{\ast }}\right)\\{}\frac{d{y}_5(t)}{dt}={Q}_5\left({y}_5(t):{\Phi}_5\right)+{Q}_{5\leftarrow 1}\left({y}_1(t):{\Phi}_{5\leftarrow 1}\right)\kern11.75em \end{array}\right. \end{equation*}

where the first term at the right-hand side of each equation is the independent genotypic value of an SNP, the second term is the epistatically induced dependent genotypic value of this SNP due to the influence of other SNPs, and third term is the pleiotropically induced dependent genotypic value of this SNP that also affects other traits. The independent genotypic values are parametrically fitted by the differential form of the logistic growth curve, whereas the dependent genotypic values nonparametrically fitted by Legendre Orthogonal Polynomials (LOP) at an appropriate order. Vectors Φ*_i_* and Φ_*i* ← *j*_ contain parameters that model parametric and nonparametric functions, respectively.

We design nine simulation scenarios by combining different numbers of time points measured (*T* = 7, 11, 15) and different residual variances (σ^2^ = 0.5, 1.0, 1.5). Under each scenario, we simulate genotypic values according to equation (1) and estimate the simulated data to reconstruct Wang’s entanglement networks. We examine and compare the statistical behavior of ODE parameter estimates under each scenario. As an example, we illustrate the estimation result under scenario 7 (Fig. S1). We find that our model can reasonably well estimate ODE parameters and, therefore, reconstruct Wang’s entanglement networks under these simulation scenarios. The precision and accuracy of parameter estimates increase with increasing measurement number and decreasing residuals (Table 1).

**Table 1:** Statistical evaluation of pleiotropic-epistatic network reconstruction under nine simulation scenarios.

σ^2^	*T*	**TPR**	**FPR**
0.5	7	0.910	0.320
	11	0.962	0.052
	15	0.990	0.036
1	7	0.760	0.370
	11	0.818	0.102
	15	0.894	0.040
1.5	7	0.654	0.342
	11	0.710	0.096
	15	0.786	0.038

We further assess the statistical power of our model by estimating true-positive rate (TPR) and false-positive rate (FPR) (Table S1). In general, the FPR is quite low under *T* = 11 and *T* = 15. The TPR is highly scenario-dependent: as the number of measurements increases and σ^2^ decreases, the TPR increases. At σ^2^ = 0.5 and *T* = 15, the TPR reaches its maximum of 0.99. We do not recommend the use of a small number of measurements when residuals are large. If residuals are unavoidably large, the number of measurements should increase as much as possible to reach a good power of pleiotropic–epistatic detection.

## Discussion

Complex traits are under the control of an unknown number of genes that work together through intricate mechanisms. Traditional quantitative genetic theory suggests that complex traits are polygenic, each gene having a small additive effect [51, 52]. Growing evidence shows that epistasis also contributes to complex traits [5, 53]. Epistasis is defined as masking of genotypes at one locus by genotypes at other loci (physiological definition usually for qualitative traits) [54] or deviation of two-locus genetic variances from the sum of the contributing single-locus genetic variances (statistical definition for quantitative traits) [55]. As genome-wide association studies develop, a more aggressive omnigenic theory emerges, suggesting that complex traits may be influenced by all genes carried by an organism [2]. Based on these classic and contemporary quantitative theories, we develop a generalized statistical mechanics framework for coalescing all possible genes into epistatic and pleiotropic networks, a genetic version of idopNetworks, a tool to capture the hidden pattern and internal working of complex systems [29, 56, 57].

This framework is the interdisciplinary combination of functional mapping, a dynamic approach for mapping the developmental pattern of genetic effects [10, 11], evolutionary game theory that strived to characterize interactions among players [21], and developmental modularity theory, a study of finding discrete units (modules) and the extent to which these modules are integrated [58, 59]. It can handle and encapsulate any number of genes into 4MidopNetworks (multilayer, multiplex, multispace, and multifunctional) that capture all genetic interactions that actually exist throughout the whole genome. This framework leverages epistasis to characterize its bidirectional, signed, and weighted properties, providing a powerful tool to detect and quantify a full amount of information about epistasis for any quantitative traits. Unlike its classic quantitative definition [51], our definition of epistasis can facilitate its translation into practical breeding schemes. For example, our model identifies a gene mediating both shoot height growth and diameter growth in woody plant, mei. A low genotypic value for genotype *AA* of this gene carried by SGTs is detected to be due to downregulation by some negative regulators. Thus, by silencing the expression of these regulates, this genotype can be induced to express more completely to achieve a greater genotypic value, making these SGTs grow fast. Similar findings in Euphrates poplar and microorganisms have been made [26, 28, 60].

As compared to previous studies, the model proposed in this article exhibits two advantages. First, it integrates epistasis and pleiotropy into Wang’s entanglement networks where these two phenomena are intertwined to mediate the growth trajectories of complex traits. Pleiotropy refers to a single gene that influences multiple phenotypic traits. A classic definition of pleiotropy is that a gene is deemed pleiotropic when its influence on at least two distinct traits is statistically significant. Nevertheless, these methods fall short of explaining how such pleiotropic genes impact the relationships and interactions among these traits. Our model can discern the direction of pleiotropy; i.e. the influence of one trait on the other and the influence of the latter on the former, both through a single gene, may be asymmetric with different magnitudes and different signs. Wang’s entanglement networks integrate both epistatic and pleiotropic effects to elucidate how one gene is regulated by other genes through epistasis and how it is influenced by multiple traits through pleiotropy. Taken together, the model provides insights into complex interactions within genetic networks and their effects on gene regulation and trait expression.

Second, classic quantitative genetic approaches can only estimate mean genetic effects of genes on complex traits from mapping or association populations, failing to characterize how epistasis is differently expressed in individual subjects. These mean parameters, expressed as additive × additive effects, additive × dominant effects, dominant × additive effects, and dominant × dominant effects [52], are instrumental for detecting genetic signals, but are limited to be utilized in practical breeding programs. Suppose additive × additive epistasis is detected to be significant. Then, one will compare phenotypic values between *AABB* and *aabb* vs *AAbb* and *aaBB*, from which to select superior ones based on their mean values. However, the efficiency of this selection cannot be enhanced by altering or editing the expression of genes. Also, each of these two-locus genotypes contains a number of individuals that may differ considerably, making it difficult to fully utilize this type of epistasis from mean values. We overcome these limitations by reconstructing Wang’s entanglement networks for individual subjects. If we limit epistasis to a single tree carrying *AA* and *BB* at two different genes, in which case we know how *AA* and *BB* are influenced by each other, then we can use gene editing to promote or repress one genotype to upregulate the expression of the other genotype. This strategy can realize the idea of precision breeding and even precision medicine when human genetic data are available.

Our model is among the first to systematically chart the genetic architecture of complex traits at the individual level, rather than at the population level, as usual in conventional quantitative genetic analysis, from widely available genetic mapping or association data. Its value is added by its capacity to dissect epistasis and its intertwined effect with pleiotropy on trait phenotypes. There is much room to improve this model, making it a more effective and efficient tool for practical use. First, genetic interactions between different loci may occur not only pairwise but also at high orders. Modeling a mix of pairwise and high-order interactions presents a meaningful way of understanding complex traits. However, a generic model for characterizing high-order interactions across a wide spectrum of biological schemes has proved to be difficult. More recently, Wu and his team introduced behavioral ecology theory to establish a universal model for estimating and detecting high-order interactions [57]. Second, the current model relies on the availability of dynamic data. To perform functional mapping from static data, we can incorporate an allometric scaling law, allowing dynamics to be extracted from static snapshots [25, 27, 59, 60]. This incorporation makes it possible to reconstruct individualized omnigenic interactome networks from static data. Third, direct associations between SNP genotypes and phenotypes ignore biological principles behind the process of genotype–phenotype linking. To fill these gaps, we need to incorporate multiomics data into Wang’s entanglement networks. Tremendous advances have been made in reconstructing interactome idopNetworks from static transcriptomic, proteomic, metabolomic, and microbiomic data [29, 30, 56, 61–63]. The integration of omics networks and Wang’s entanglement networks will not be technically difficult, but can be expected to provide a more comprehensive understanding of complex trait genetics.

Our model is the extension of our previous population-based network inference, which has been validated empirically and experimentally [26, 28], to the individual level. Individual-based networks can characterize how genes act and interact with each other in individual subjects, a piece of information useful for designing polygenic editing toward trait improvement for target individuals. Consider an individual #1 in Fig. 3. We assume that SNP 2′ genotype aa is positively associated with this individual’s stress resistance. Although this genotype is promoted by SNP 1’s genotype *AA*, it is inhibited by SNP *m*’s *AA*. Thus, to improve this individual stress resistance through SNP 2, one may repress the transcriptional expression of SNP *m*’s *AA* by polygenic editing.

We perform computer simulation to validate the utility and usefulness of our extended model. In conjunction with previous extensive simulation and experimental results, we are confident that the new model can provide biologically meaningful interpretation of complex trait genetics. The application of the new model to analyzing practical mapping data for an ornamental woody plant, mei, has further justified its biological value for understanding genetics. In summary, our model could hold great promise to change our view into quantitative genetics and make this old discipline a renaissance of practical application in the era of big data.

## Methods

Figure 3 is a flowchart of reconstructing idopNetworks from a genome-wide mapping or association study. Functional mapping (FunMap) is a first step to estimate genotype-dependent growth parameters and draw genotype-dependent growth curves [10] (Supplementary Text). An individual in the population carries one and only one genotype at an SNP. Each individual is assigned a series of time-dependent genotypic values at an SNP using the estimated growth parameters of the genotype carried by this individual. Below is an illustration of the basic principle for idopNetwork reconstruction.

### MODE modeling of evolutionary game theory

The time-varying genotypic values of an individual at each SNP are estimated by FunMap. Let ${\hat{y}}_{s_l}(t)$ denote the estimated value of genotype *l* at a given SNP *s*, carried by this individual, at time point *t*. For an individual, its different genotypes at different loci may epistatically interact with each other to determine its overall genotypic value. The interaction between different loci can be interpreted through the lens of evolutionary game theory. The genotypic value of an SNP for an individual is determined by this SNP’s intrinsic allelic capacity and the influence of other loci. The intrinsic capacity of a SNP produces a so-called independent genotypic component (formed when it is assumed to be independent from other loci) and the influence of other loci produces a dependent genotypic component. To separate these two components, we derive a system of mODE by assuming all genes in this individual to act as an interactive complex system [24, 26, 28]. These mODEs are written as


(2) \begin{equation*} \frac{\ d{\hat{y}}_{s_l}(t)\ }{dt}={Q}_{s_l}\left({\hat{y}}_{s_l}(t)\ \right)+\sum_{s^{\prime }=1,{s}^{\prime}\ne s}^m{Q}_{s_l\leftarrow{s}_{l^{\prime}}^{\prime }}\left({\hat{y}}_{s_{l^{\prime}}^{\prime }}(t)\right)\kern2.5em \end{equation*}

where the genotypic value at SNP *s* is decomposed into its independent genotypic component ${Q}_{s_l}\left({\hat{y}}_{s_l}(t)\ \right)$ and dependent genotypic component $\sum_{s^{\prime }=1,{s}^{\prime}\ne s}^m{Q}_{s_l\leftarrow{s}_{l^{\prime}}^{\prime }}\left({\hat{y}}_{s_{l^{\prime}}^{\prime }}(t)\right)$, with the former expressed as the self-regulation function of this SNP’s genotypic value and the latter expressed as the coregulation function of genotypic values at all loci, except for SNP *s* (e.g. genotype ${s}_{l^{\prime}}^{\prime }$ at SNP ${s}^{\prime }$). The independent genotypic value component can be described by a differential growth equation, but it can be generally smoothened by a nonparametric approach, such as LOP [64]. The dependent component curves generally have no explicit form, which is thus smoothened by an LOP-like nonparametric approach. Consider *R*-order basis LOP functions at time *t*, ${p}_0(t)=1,$  ${p}_1(t)=t$, ${p}_2(t)=\frac{3{t}^2-1}{2}$, . . ., ${p}_R(t)=\frac{1}{2^RR!}\frac{d^R}{d{t}^R}\left[{\left({t}^2-1\right)}^R\right]$, where time *t* is rescaled to the interval [−1,1]. Let ${\mathbf{p}}_{s_l}(t)=\left({p}_0(t),{p}_1(t),\dots, {p}_{R_s}(t)\right)$ and ${\mathbf{p}}_{s_{l^{\prime}}^{\prime }}(t)=\left({p}_0(t),{p}_1(t),\dots, {p}_{R_{s^{\prime }}}(t)\right)$ denote the vectors of basis LOP values at order ${R}_s$ and ${R}_{s^{\prime }}$ for the independent component and the dependent component, respectively. Let ${\mathbf{u}}_{s_l}=\left({u}_{s_l0},{u}_{s_l1}\dots, {u}_{s_l{R}_s}\right)$ and ${\mathbf{u}}_{s_l\leftarrow{s}_{l^{\prime}}^{\prime }}=\left({u}_{s_l\leftarrow{s}_{l^{\prime}}^{\prime }0},{u}_{s_l\leftarrow{s}_{l^{\prime}}^{\prime }1}\dots, {u}_{s_l\leftarrow{s}_{l^{\prime}}^{\prime }{R}_{s^{\prime }}}\right)$ denote a vector of values of model parameters corresponding to the basis LOP values at orders ${R}_s$ and ${R}_{s^{\prime }}$, which determine the independent and dependent components, respectively. After LOP transformation, the mODE of equation (2) is rewritten as


(3) \begin{equation*} \frac{\ d{\hat{y}}_{s_l}(t)}{dt}={\mathbf{u}}_{s_l}{\mathbf{p}}_{s_l}^{\mathrm{T}}(t){\hat{y}}_{s_l}(t)+\sum_{s^{\prime }=1,{s}^{\prime}\ne s}^m{\mathbf{u}}_{s_l\leftarrow{s}_{l^{\prime}}^{\prime }}{\mathbf{p}}_{s_{l^{\prime}}^{\prime}}^{\mathrm{T}}(t)\ {\hat{y}}_{s_{l^{\prime}}^{\prime }}(t) \end{equation*}

where T is the vector transpose. By taking the integral of the smoothed mODE, we express the genotypic value of SNP *s* for an individual as


(4) \begin{align*}\begin{aligned} {z}_{s_l}(t)&=\int \frac{\ d{\hat{y}}_{s_l}(t)}{dt}+{e}_{s_l}(t)\\ &={\mathbf{u}}_{s_l}{\mathbf{q}}_{s_l}^{\mathrm{T}}(t){\hat{y}}_{s_l}(t)+\!\sum_{s^{\prime }=1,{s}^{\prime}\ne s}^m\!{\mathbf{u}}_{s_l\leftarrow{s}_{l^{\prime}}^{\prime }}{\mathbf{q}}_{s_{l^{\prime}}^{\prime}}^{\mathrm{T}}(t){\hat{y}}_{s_{l^{\prime}}^{\prime }}(t)+{e}_{s_l}(t)\end{aligned} \end{align*}

where **q**. is the antiderivative transformation of **p**., with the values of model parameters being unchanged, and ${e}_{s_l}(t)$ is the residual at time *t*, normally distributed as (0, ${\sigma}_{s_l}^2$), which cannot be explained by the independent and dependent components.

We formulate a likelihood to obtain the maximum likelihood estimates (MLE) of unknown parameters that model the independent and dependent components. We assume that ${\mathbf{z}}_{s_l}=\left({z}_{s_l}\left({t}_1\right),\dots, {z}_{s_l}\left({t}_T\right)\right)$ follows a multivariate normal distribution $f\Big({\mathbf{z}}_{s_l}:{\boldsymbol{\mathrm{\mu}}}_{s_l},{\boldsymbol{\Sigma}}_{s_l}$), with mean vector ${\boldsymbol{\mathrm{\mu}}}_{s_l}=\left({\mu}_{s_l}\left({t}_1\right),\dots, {\mu}_{s_l}\left({t}_T\right)\right)$ modeled by the integral of the mODE in equation (4) and covariance matrix ${\boldsymbol{\Sigma}}_{s_l}$ modeled by the first-order structured antedependence (SAD(1)). Statistical or machine learning approaches can be deployed to estimate LOP parameters that model the independent and dependent components and SAD (1) parameters that model the structure of the covariance matrix [36, 65]. After the MLEs of the independent genotypic and dependent genotypic components are estimated, we use graph theory to encode these components as nodes and edges, respectively, into mathematical genetic control networks (Fig. 8). These so-called individualized dynamic omnidirectional polynomial (idop) networks are fully informative (in terms of their capacity to capture bidirectional, signed, and weighted interactions), dynamic (because of the independent and dependent components as a function of time), omnidirectional (with ability to cover all possible existing interactions), and individualized (networks reconstructed for individual samples). Also, such idopNetworks can be reconstructed for each individual (Fig. 8). An information criterion, such as the Akaike Information Criterion (AIC) or the Bayesian Information Criterion (BIC), is used to determine an optimal order of LOP for curve smoothing.

### Reconstructing sparse networks

As the number of SNPs increases, it is computationally challenging to reconstruct an idopNetwork covering all SNPs. This issue can be overcome through the lens of two universal theories. Developmental modularity theory states that complex systems are broken down into multiple distinct modulars in which parts are linked more closely with each other than with those from other modules [58]. This theory suggests that a large network can be divided into different subnetworks or network communities by a statistical clustering approach [66]. Sparsity theory suggests that any part of a complex system is not necessarily linked to all other parts because a fully interconnected system is vulnerable to environmental perturbations [67]. In other words, one part may be linked to a small set of other parts to form a sparse network.

Considering developmental modularity theory, we implement a bottom-up approach for subnetwork detection. This approach aims to classify all SNPs into multiple distinct modules based on the similarity of the time-varying pattern of genetic effects. Let ${\hat{\mathbf{y}}}_{s_l}=\left({\hat{y}}_{s_l}\left({t}_1\right),\dots, {\hat{y}}_{s_l}\left({t}_T\right)\right)$ denote a vector of genotypic values for a given individual carrying genotype *l* at SNP *s* (*s* = 1, . . ., *p*). Under the assumption that *p* SNPs are classified into *K* clusters, an SNP can be attributed to one of *K* possible clusters with a frequency (prior probability) of ${\pi}_k\ \left(k=1,\dots, K\right)$. To do so, we implement functional clustering [45, 68] by formulating a mixture likelihood of genotypic values at all SNPs ($\hat{\mathbf{y}}$), expressed as


(5) \begin{equation*} L\left(\hat{\mathbf{y}}\right)=\prod_{s=1}^p\left({\pi}_1{f}_1\left({\hat{\mathbf{y}}}_{s_l}:{\boldsymbol{\mathrm{\mu}}}_1,\boldsymbol{\Sigma} \right)+\dots +{\pi}_K{f}_K\left({\hat{\mathbf{y}}}_{s_l}:{\boldsymbol{\mathrm{\mu}}}_L,\boldsymbol{\Sigma} \right)\right)\kern7.75em \end{equation*}

where ${f}_k\left({\hat{\mathbf{y}}}_{s_l}:{\boldsymbol{\mathrm{\mu}}}_k,\boldsymbol{\Sigma} \right)$ is the longitudinal multivariate normal density function of ${\hat{\mathbf{y}}}_{s_l}$ for SNP *l* belonging to cluster *k*, with mean vector ${\boldsymbol{\mathrm{\mu}}}_k$ and covariance matrix $\boldsymbol{\Sigma}$. According to the principle of functional clustering, we use a function to describe time-varying genotypic means within mean vectors and an autoregressive model to structure the covariance matrix. In this case, we implement the growth equation (4) to define genotypic values ${\mu}_k(t)$ within mean vector *k* and SAD (1) to model time-varying variances and covariances.

We implement a hybrid of the EM algorithm and the Nelder–Mead (simplex) algorithm to estimate the MLEs of all parameters that model mean-covariance structures by deriving two successive steps. In the E step, we calculate the posterior probability with which SNP *s* is assigned to cluster *k* given its genotypic values, expressed as


(6) \begin{equation*} {\Pi}_{k\mid s}=\frac{\pi_k{f}_k\left({\hat{\mathbf{y}}}_{s_l}:{\boldsymbol{\mathrm{\mu}}}_k,\boldsymbol{\Sigma} \right)}{\pi_1{f}_1\left({\hat{\mathbf{y}}}_{s_l}:{\boldsymbol{\mathrm{\mu}}}_1,\boldsymbol{\Sigma} \right)+\dots +{\pi}_K{f}_K\left({\hat{\mathbf{y}}}_{s_l}:{\boldsymbol{\mathrm{\mu}}}_L,\boldsymbol{\Sigma} \right)}.\kern9.75em \end{equation*}

In the M step, we estimate the model parameters that maximize the likelihood of equation (5) by using the Nelder–Mead (simplex) algorithm. Note that the determinant and inverse of $\boldsymbol{\Sigma}$ have explicit forms that can be implemented into the M step to enhance computational efficiency. The E and M steps are iterated until the estimates of the parameters converge to stable values. To determine an optimal number of clusters, we calculate the AIC or BIC values as criteria. After the MLEs of model parameters are obtained, we plug in these values into equation (6) to calculate the posterior probability of each SNP. An SNP is assigned to a cluster in which case the posterior probability of this SNP belonging to this cluster is the largest.

Considering sparsity theory, we implement variable selection to choose a subset of the most significant predictors associated with a given SNP *s*, based on a regression model described by equation (4). Regularization-based variable selection has proven to be powerful for producing accurate prediction while selecting a subset of important factors, including L_1_-norm LASSO and L_2_-norm ridge and L_1_/L_2_-norm group LASSO [69]. We implement the convex optimization technique of L_1_/L_2_ regularization for variable selection, expressed as


(7) \begin{align*} {\mathbf{u}}^{\ast }=&\ \underset{\mathbf{u}}{\mathrm{argmin}}{\left\Vert{z}_{s_l}(t)\!-\!{u}_{s_l}t-{\mathbf{u}}_{s_l}{\mathbf{q}}_{s_l}^{\mathrm{T}}(t){\hat{y}}_{s_l}(t)\notag \vphantom{\underset{\mathbf{u}}{\mathrm{argmin}}{\left\Vert{z}_{s_l}(t)\!-\!{u}_{s_l}t-{\mathbf{u}}_{s_l}{\mathbf{q}}_{s_l}^{\mathrm{T}}(t){\hat{y}}_{s_l}(t)-\sum_{s^{\prime }=1,{s}^{\prime}\ne s}^m{\mathbf{u}}_{s_l\leftarrow{s}_{l^{\prime}}^{\prime }}{\mathbf{q}}_{s_{l^{\prime}}^{\prime}}^{\mathrm{T}}(t){\hat{y}}_{s_{l^{\prime}}^{\prime }}(t)\right\Vert}_2^2} \right.} \\&{\left. -\sum_{s^{\prime }=1,{s}^{\prime}\ne s}^m{\mathbf{u}}_{s_l\leftarrow{s}_{l^{\prime}}^{\prime }}{\mathbf{q}}_{s_{l^{\prime}}^{\prime}}^{\mathrm{T}}(t){\hat{y}}_{s_{l^{\prime}}^{\prime }}(t)\right\Vert}_2^2 \\ & +\lambda \sum_{s^{\prime }=1,{s}^{\prime}\ne s}^m\sqrt{R_{s^{\prime }}}\ {\left\Vert{\mathbf{u}}_{s_l\leftarrow{s}_{l^{\prime}}^{\prime }}\right\Vert}_2\kern8.75em \notag \end{align*}

where $\lambda$ is a regularization parameter and ${R}_{s^{\prime }}$ is the order of LOP associated with SNP ${s}^{\prime }$. Group LASSO on equation (4) can find a small subset of SNPs that are associated with the focal SNP ${s}^{\prime }$. Using these small set of SNPs, a sparse idopNetwork can be reconstructed.

By combining functional clustering and group LASSO, we classify all SNPs into multiple clusters and, if needed, classify all SNPs from a cluster into multiple subclusters. This procedure is repeated until the number of SNPs within a unit reaches a number by which each SNP can establish stable relationships with other SNPs. To reconstruct a sparse SNP–SNP interaction network for such a unit, group LASSO is implemented. At the end, we will reconstruct a multilayer, multiplex, multiscale, and multifunctional idopNetwork for each individual, which allows these networks to be compared among different individuals.

## Supplementary Material

Web_Material_uhaf345

## Data Availability

The data and code are available at https://github.com/ChangjianFa/BIMSA_IdopNetwork.
