# Supplementary material for: An omnigenic interactome model to chart the genetic architecture of individual plants
Source: Hortic Res. 2025 Dec 16;13(3):uhaf345. doi: 10.1093/hr/uhaf345 (PMC12977963; doi:10.1093/hr/uhaf345)
Supplement: Web_Material_uhaf345 [file web_material_uhaf345.docx]

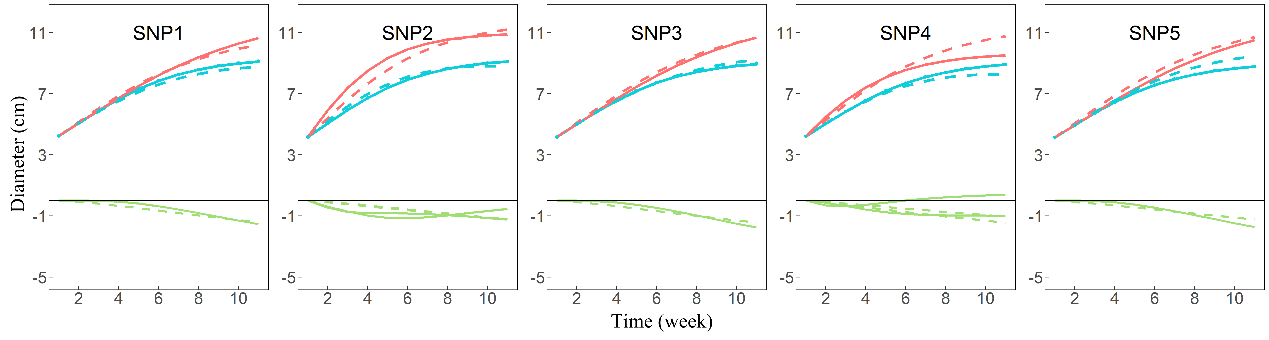


**Figure S1.** The estimates of time-varying genotypic effect curves (dashed line) under simulation scenario *T* = 15 and σ^2^ = 0.5, in a comparison with true genotypic effects curves (solid line), for a 5-SNPs interaction network. The overall curve (blue) is decomposed into its underlying independent curve (red) and dependent curves (green).

**Table 1**. Statistical evaluation of pleiotropic-epistatic network reconstruction under nine simulation scenarios.

| **σ^2^** | *T* | **TPR** | **FPR** |
| --- | --- | --- | --- |
| 0.5 | 7 | 0.910 | 0.320 |
|  | 11 | 0.962 | 0.052 |
|  | 15 | 0.990 | 0.036 |
| 1 | 7 | 0.760 | 0.370 |
|  | 11 | 0.818 | 0.102 |
|  | 15 | 0.894 | 0.040 |
| 1.5 | 7 | 0.654 | 0.342 |
|  | 11 | 0.710 | 0.096 |
|  | 15 | 0.786 | 0.038 |
